# The next frontier in immunotherapy: potential and challenges of CAR-macrophages

**DOI:** 10.1186/s40164-024-00549-9

**Published:** 2024-08-05

**Authors:** Jing Li, Ping Chen, Wenxue Ma

**Affiliations:** 1 The Affiliated Hospital of Qingdao University, Qingdao, 266003 Shandong China; 2 Fujian Institute of Hematology, Fujian Provincial Key Laboratory of Hematology, Union Hospital, Fujian Medical University Fuzhou, Fujian, 350001 China; 3 Sanford Stem Cell Institute, Moores Cancer Center, University of California San Diego, CA 92093 La Jolla, USA

**Keywords:** CAR macrophage (CAR-MΦ), Immunotherapy, Tumor Microenvironment (TME), Combination therapies, Clinical trials

## Abstract

Chimeric antigen receptor macrophage (CAR-MΦ) represents a significant advancement in immunotherapy, especially for treating solid tumors where traditional CAR-T therapies face limitations. CAR-MΦ offers a promising approach to target and eradicate tumor cells by utilizing macrophages’ phagocytic and antigen-presenting abilities. However, challenges such as the complex tumor microenvironment (TME), variability in antigen expression, and immune suppression limit their efficacy. This review addresses these issues, exploring mechanisms of CAR-MΦ action, optimal construct designs, and interactions within the TME. It also delves into the ex vivo manufacturing challenges of CAR-MΦ, discussing autologous and allogeneic sources and the importance of stringent quality control. The potential synergies of integrating CAR-MΦ with existing cancer therapies like checkpoint inhibitors and conventional chemotherapeutics are examined to highlight possible enhanced treatment outcomes. Furthermore, regulatory pathways for CAR-MΦ therapies are scrutinized alongside established protocols for CAR-T cells, identifying unique considerations essential for clinical trials and market approval. Proposed safety monitoring frameworks aim to manage potential adverse events, such as cytokine release syndrome, crucial for patient safety. Consolidating current research and clinical insights, this review seeks to refine CAR-MΦ therapeutic applications, overcome barriers, and suggest future research directions to transition CAR-MΦ therapies from experimental platforms to standard cancer care options.

**Supplementary Information:**

The online version contains supplementary material available at 10.1186/s40164-024-00549-9.

## Highlights

CAR-MΦ offers an innovative approach to treating solid tumors, addressing the limitations of traditional CAR-T therapies.

CAR-MΦ eliminates tumor cells and boosts other immune cells’ effectiveness.

Strategies are being t developed to improve CAR-MΦ targeting and cancer cell eradication.

CAR-MΦ is combined with other treatments to enhance overall efficacy.

Challenges and safety concerns, including side effects of CAR-MΦ therapies, are Beijing addressed.

**Supplementary Information:**

The online version contains supplementary material available at 10.1186/s40164-024-00549-9.

## Background

Immunotherapy has revolutionized cancer treatment by leveraging the body’s immune system to detect and eradicate malignant cells [1]. The field has seen substantial advancements over the past decade with the emergence of immune checkpoint inhibitors, cancer vaccines, and adoptive cell transfer therapies, each contributing to a significant shift in oncological therapeutic strategies [2–4]. Among these innovations, Chimeric Antigen Receptor T-cells (CAR-T) and Natural Killer cells (CAR-NK) represent breakthrough therapies [5, 6]. CAR-T cell therapy has shown exceptional efficacy in treating hematologic malignancies by reprogramming T cells to target and destroy tumor cells specifically [5]. Although CAR-NK therapies are still in the experimental stages, they have shown promise in offering similar therapeutic benefits but with potentially fewer adverse effects, such as cytokine release syndrome (CRS) and graft-versus-host disease (GVHD), which are more common in CAR-T cell treatments [7–10].

However, applying these cellular therapies to solid tumors has been fraught with challenges [5, 11]. The primary obstacles include the immunosuppressive nature of the tumor microenvironment (TME), the heterogeneity of tumor antigens, and physical barriers that restrict cellular infiltration into tumors [12–14]. These challenges have sparked significant debate and exploration within the research community, as there is a consensus that overcoming these barriers could unlock new therapeutic potentials for solid tumors [15, 16].

CAR macrophages (CAR-MΦ) may offer strategic benefits in reshaping the TME and triggering a comprehensive immune response due to their phagocytic nature and antigen-presentation capabilities, which could lead to more sustained tumor control [17]. This contentious backdrop has led to exploring CAR-MΦ as a novel therapeutic avenue. Macrophages, known for their roles in tissue homeostasis, inflammation, and immune surveillance, are engineered to express chimeric antigen receptors [18, 19]. This approach aims to harness their inherent phagocytic nature and ability to modulate the TME, positioning them as potentially effective agents in combating solid tumors [20–22]. Despite the theoretical benefits, considerable controversy exists regarding the efficacy, safety, and practical application of CAR-MΦ [22, 23]. Current knowledge is limited, particularly in direct clinical outcomes and mechanistic understanding of CAR-MΦ actions within varied TMEs [13, 24, 25].

## Structural details and potential synergy with checkpoint inhibitors

### Structural details of CAR-MΦ

The structure of CAR-MΦ is crucial for their function and therapeutic efficacy. CAR-MΦ are typically engineered to express CARs that include an extracellular antigen-binding domain derived from an antibody’s single-chain variable fragment (scFv). This domain is linked to intracellular signaling domains, which are crucial for activating macrophages upon antigen engagement [26]. These signaling domains often include co-stimulatory molecules such as CD28 or 4-1BB, which enhance macrophage survival, proliferation, and phagocytic efficacy [8, 19].

### Potential synergy with checkpoint inhibitors

CAR-MΦ therapy’s potential synergy with checkpoint inhibitors is a promising avenue for enhancing anti-tumor efficacy. Checkpoint inhibitors, such as those targeting PD-1/PD-L1 and CTLA-4 pathways, block inhibitory signals that dampen immune responses, thereby reactivating T cells to attack tumors [27, 28]. Combining CAR-MΦ with checkpoint inhibitors aims to overcome the immunosuppressive TME, thus enhancing the overall therapeutic outcome [29]. Recent studies have demonstrated the synergy between CAR-MΦ and checkpoint inhibitors. Yang et al. found that CAR-MΦ engineered with anti-PD-L1 scFv showed enhanced anti-tumor efficacy in preclinical models [30]. Harrasser et al. reported that localized delivery of an anti-PD-1 scFv boosts the antitumor activity of ROR1 CAR-T cells in triple-negative breast cancer (TNBC) [31]. Li et al. showed that combining CAR-MΦ with anti-CTLA-4 therapy enhances tumor cell phagocytosis and promotes a robust immune response [32].

## Clinical efficacy and safety

### Clinical evidence

The clinical exploration of CAR-MΦ is rapidly progressing, particularly for solid tumors where traditional CAR-T therapies face significant challenges [16, 18, 33, 34]. Current clinical trials primarily focus on assessing CAR-MΦ’s efficacy in reducing tumor mass and evaluating their safety for patients who have exhausted conventional treatments. Initial findings show CAR-MΦ can effectively localize to and persist within tumor sites, providing promising insights for ongoing and future research [18, 35]. However, comprehensive outcome data and extended follow-up are needed to understand CAR-MΦ’s long-term efficacy and safety [36].

One ongoing clinical trial, NCT04660929, is a Phase I study evaluating CAR-MΦ for treating HER2-overexpressing solid tumors. This trial includes patients with various HER2-positive cancers, such as breast, bladder, and lung cancers, and focuses on assessing the safety and preliminary efficacy of CAR-MΦ. Initial findings have shown that CAR-MΦ therapy is safe and well-tolerated, with some indications of anti-tumor activity, including tumor regression and enhanced T-cell infiltration at the tumor site [37]. However, extended follow-up is necessary to determine this therapeutic approach’s long-term benefits and potential risks.

Preclinical studies have demonstrated CAR-MΦ’s unique capabilities, particularly their ability to modulate the complex TME, supporting immune-mediated tumor destruction [17, 19]. These studies have shown that CAR-MΦ not only directly attacks tumor cells but also transforms the typically suppressive TME into a more active, anti-tumor environment [34, 38]. By secreting pro-inflammatory cytokines and chemokines, CAR-MΦ recruits and activates other immune cells, suggesting a significant role in enhancing the efficacy of combination immunotherapies [30, 39].

### Safety profile

The development and advancement of CAR-MΦ therapies bring promising therapeutic opportunities and significant safety considerations that mirror those observed with CAR-T cell therapies [40]. Both are known for their potential to cause severe adverse effects such as CRS and neurotoxicity due to their robust cytokine production capabilities [41, 42]. However, macrophages’ intrinsic regulatory functions in managing inflammation suggest CAR-MΦ might control cytokine release more effectively, underscoring the need for research into their unique cytokine dynamics [43, 44].

CRS is a critical concern previously well-documented in CAR-T therapy, manifesting as a systemic inflammatory response that leads to life-threatening [5, 45, 46]. Similar risks are possible with CAR-MΦ therapies [19]. However, the distinct role of macrophages in cytokine regulation may result in different CRS dynamics, necessitating tailored strategies for anticipation, monitoring, and management [34, 42]. Recent studies suggest that engineering CAR-MΦ to express IL-10 can mitigate CRS while maintaining anti-tumor efficacy [19, 47].

Another safety concern is hemophagocytic lymphohistiocytosis (HLH) and macrophage activation syndrome (MAS), involving excessive immune activation and organ damage [48, 49]. This is particularly relevant to CAR-MΦ therapies due to their role in these conditions [50, 51]. Ongoing vigilance in monitoring engineered cell activation and inflammatory responses is crucial to prevent HLH/MAS.

Significant gaps remain in understanding how risks from CAR-T therapies translate to CAR-MΦ therapies [38, 52]. Questions include how CAR-MΦ modulates cytokine output and whether this modulation can be controlled to prevent adverse effects like CRS [33, 39, 53]. Furthermore, the long-term implications of CAR-MΦ therapy, especially concerning potential chronic inflammation or immune dysregulation, and the specificity of CAR-MΦ targeting to minimize off-target effects, need further exploration [54].

Comprehensive preclinical and clinical research on the unique safety dynamics of CAR-MΦ therapies is essential [17, 19, 34]. Developing accurate monitoring protocols and effective management strategies for potential adverse effects is imperative. Moreover, a deeper understanding of CAR-MΦ interactions with the immune system is crucial for maximizing therapeutic potential, mitigating risks, and integrating CAR-MΦ therapies into clinical oncology practice [51, 55].

**Fig. 1:**
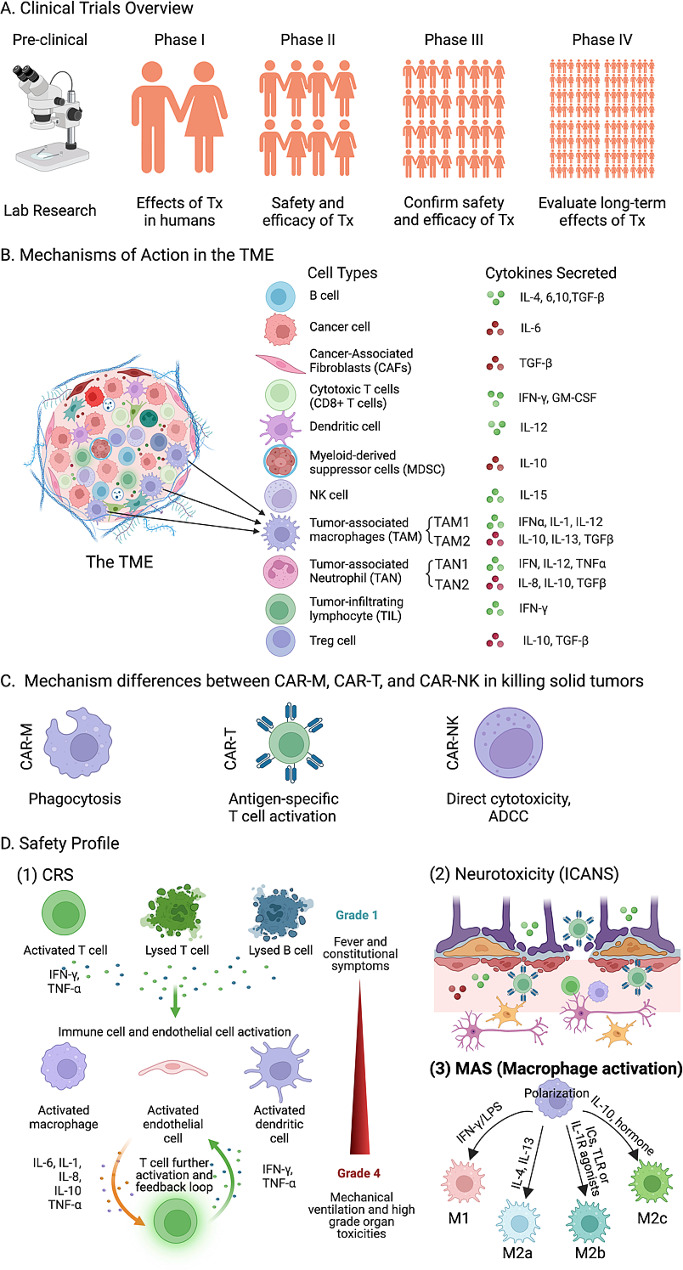
Overview of CAR Cell Therapies: Clinical Trials, TME Interaction, and Safety Profiles. (**A**) Clinical Trials Overview: This panel illustrates the stages of clinical trial progression for CAR cell therapies, from pre-clinical lab research to Phase IV, detailing the evaluation of treatment effects in humans, safety and efficacy assessments, and the long-term impact of treatments. (**B**) Mechanisms of Action in the TME: Diagram displaying the diverse cellular composition and cytokine environment of the TME. The relationships and influences between different cell types and secreted cytokines are highlighted, showing the dynamic interactions within the TME that impact therapy outcomes. (**C**) Mechanism Differences between CAR-MΦ, CAR-T, and CAR-NK in Killing Solid Tumors: This segment compares the functional approaches of CAR-MΦ, CAR T-cells, and CAR natural killer cells (CAR-NK in combating solid tumors, emphasizing the unique mechanisms like phagocytosis by CAR-MΦ, antigen-specific T cell activation by CAR-T, and direct cytotoxicity along with antibody-dependent cellular cytotoxicity (ADCC) by CAR-NK. (**D**) Safety Profile: Outlines the critical safety concerns associated with CAR cell therapies, including CRS, neurotoxicity (ICANS), and macrophage activation syndrome (MAS). The panel describes the progression of CRS symptoms from mild to severe, details the cellular and molecular processes involved in ICANS, and explains the various macrophage polarization states in MAS, along with their associated cytokines

Figure 1 below provides a detailed representation of the essential progression, intricate dynamics within the TME, and critical safety considerations associated with CAR cell therapies.

### Recommendations for further research

As the potential of CAR-MΦ therapies unfolds, a comprehensive understanding of their clinical implications, particularly regarding safety and efficacy, is essential [39, 56]. Insights from current clinical trials are invaluable, yet they also highlight substantial gaps in understanding, especially concerning long-term impacts and broader applicability across various cancer types [18, 37].

Expanding the scope of clinical trials is crucial for thoroughly assessing the therapeutic potential and safety profile of CAR-MΦ across a broader spectrum of cancer types [57]. This expansion involves increasing the number of trials and including a diverse range of participants to explore how different demographics respond to CAR-MΦ therapy. Additionally, investigating CAR-MΦ interactions with other cancer treatments, such as chemotherapy or immunotherapy, could provide insights into potential synergistic effects or complications [17, 58]. Experimenting with various CAR designs and administration strategies could also optimize the balance between efficacy and safety, improving the overall outcomes of CAR-MΦ therapies [56, 59].

There is also a pressing need for long-term follow-up studies to understand the sustained impact of CAR-MΦ treatments on patients. These studies are critical for evaluating the durability of therapeutic benefits, potential late-onset adverse effects, and overall quality of life post-treatment [60]. Understanding the long-term effects of CAR-MΦ therapy on the immune system, including possible impacts on immune memory and susceptibility to infections or other diseases, is vital [34, 61].

Despite promising advances in CAR-MΦ research, several significant controversies and unanswered questions remain. Debates continue over the best strategies for engineering and administering CAR-MΦ, focusing on maximizing efficacy while minimizing risks. The challenges of defining the optimal configuration of CAR constructs and the best delivery methods are compounded by significant regulatory and ethical questions, particularly regarding patient consent processes and trial inclusion criteria [62, 63].

To fully harness the therapeutic potential of CAR-MΦ and ensure their safe integration into clinical oncology, it is essential to expand clinical trials and conduct detailed long-term follow-up studies [64]. These efforts are crucial for filling current knowledge gaps and addressing broader controversies and challenges in the field. As research continues, these focused efforts will help pave the way for CAR-MΦ therapies to transition from experimental treatments to established options within the oncological arsenal, ensuring they are both practical and safe for clinical use [65].

## Comparison with other CAR cells

### CAR-T cells

CAR-T therapy has revolutionized the treatment of hematological malignancies such as acute lymphoblastic leukemia (ALL) and diffuse large B-cell lymphoma (DLBCL) [64, 66]. This therapy targets and eliminates cancer cells with specific antigens, demonstrating significant efficacy. However, extending CAR-T therapy’s success to solid tumors has proven complex, revealing intrinsic limitations that underscore the challenges of applying this therapy across diverse oncological applications [67, 68].

TME in solid tumors presents formidable physical and immunological barriers to CAR-T therapy [67, 69]. While CAR-T cells are highly effective in blood cancers, their application in solid tumors has not met with the same success due to the TME’s complexity, which includes immunosuppressive cells, inhibitory cytokines like TGF-β and IL-10, and physical barriers that restrict CAR-T cell penetration and persistence [6, 13, 14]. Strategies to enhance CAR-T cell infiltration and survival within these hostile environments remain a significant focus of ongoing research [67, 70].

### CAR-NK cells

CAR Natural Killer (CAR-NK) cells are rapidly emerging as a promising frontier in adoptive cell therapies, leveraging the innate capabilities of NK cells to recognize and eliminate malignant cells without prior sensitization [71]. By engineering these cells to express specific antigen receptors, researchers have expanded their targeting capabilities and enhanced their natural cytotoxic abilities, which include direct induction of cell death and release of cytolytic granules containing perforin and granzymes [72]. Additionally, CAR-NK cells can mediate ADCC, enhancing their utility against tumors that express specific antigens [73].

The clinical applications of CAR-NK cells have shown promising results, particularly in treating hematologic malignancies such as leukemia and lymphoma [74]. However, translating these successes to solid tumors presents substantial challenges. The immunosuppressive TME in solid tumors can significantly inhibit CAR-NK cell function and persistence. In contract, the heterogeneity of tumor antigens and the potential for antigen escape pose additional hurdles to their clinical effectiveness [10, 75, 76].

When compared with CAR-NK cells with CAR-MΦ, both modalities encounter similar challenges in solid tumors, particularly concerning immunosuppressive TME [10, 77]. However, CAR-NK cells may possess inherent advantages due to their cytotoxic mechanisms and ability to engage in ADCC, potentially providing a more robust and immediate response to tumor cells [78, 79].

### CAR-MΦ

CAR-MΦ is making significant strides in adoptive cell therapy by utilizing the innate biological functions of macrophages to combat cancer [19, 30]. These engineered immune cells exploit macrophages’ natural phagocytic and antigen-presenting abilities, offering a novel dimension in cancer treatment, particularly effective against solid tumors [19, 34]. The dual functionality of CAR-MΦ allows them to reduce tumor mass by engulfing and digesting tumor cells and to process and present antigens, thereby catalyzing a broader systemic immune response against the tumor [34].

Beyond their immediate impact on cancer cells, CAR-MΦ is adept at navigating and modulating the complex and often hostile TME [33, 80]. Their inherent migratory and infiltrative capabilities enable them to overcome physical barriers within the TME that typically shield tumor cells from immune attacks [13, 81]. Once inside the TME, CAR-MΦ can disrupt the local immunosuppressive conditions by secreting pro-inflammatory cytokines and chemokines, making the environment more amenable to immune-mediated attack [36, 82].

Despite these significant advantages, CAR-MΦ faces several critical challenges that limit their broader application. The field widely recognizes the difficulty in identifying specific targets on tumor cells that can be consistently recognized by the engineered receptors on CAR-MΦ, given the heterogeneity of tumor cells and the potential for antigen escape mechanisms [17, 19]. This challenge underscores the ongoing debate over the specificity and efficacy of CAR-MΦ targeting and the need for continued research into universal tumor markers that CAR-MΦ can reliably target [83].

Moreover, like other CAR therapies, CAR-MΦ is at risk of inducing CRS, a severe side effect arising from cytokine’s rapid release into the bloodstream [19]. This safety concern mirrors those associated with CAR-T therapies and fuels further debate on the clinical viability of CAR-MΦ [19]. Addressing this risk necessitates careful CAR construct design and strategies to control CAR-MΦ activity once administered to patients [17, 39].

**Table 1:** Advantages and limitations of CAR-T, CAR-NK, and CAR-MΦ therapies

Therapy Type	Advantages	Limitations
CAR-T	1 High Specificity: Highly effective in targeting specific antigens, particularly in hematologic cancers. 2 Established Protocols: Well-established clinical protocols and substantial clinical data.	1 TME Challenges: Limited efficacy in solid tumors due to immunosuppressive TME and T-cell exhaustion. 2 CRS and Neurotoxicity: Significant risks of severe adverse effects like CRS and neurotoxicity.
CAR-NK	1 Innate Cytotoxicity: Ability to kill tumor cells without prior sensitization. 2 Lower GVHD Risk: Lower risk of graft-versus-host disease (GVHD) compared to CAR-T cells.	1 Persistence and Expansion: Challenges in ensuring the persistence and expansion of CAR-NK cells within the TME. 2 Efficacy in Solid Tumors: Like CAR-T cells, CAR-NK cells face significant hurdles in solid tumors due to the TME.
CAR-MΦ	1 TME Modulation: CAR-MΦ can effectively remodel the TME to a more pro-inflammatory state, which is advantageous in solid tumors. 2 Antigen Presentation: Enhances the activation of T cells and overall immune response against tumors.	1 Phagocytic Efficiency: The efficiency of tumor cell engulfment in an immunosuppressive TME can be variable. 2 CRS: Potential risk of CRS, like CAR-T cells, though macrophages have intrinsic regulatory functions that might mitigate this risk.

To encapsulate the distinct characteristics and challenges faced by CAR-T, CAR-NK, and CAR-MΦ therapies in solid tumors, Table 2 offers a comparative overview, highlighting their respective advantages and limitations.

**Table 2:** Differences between CAR-MΦ, CAR-T, and CAR-NK therapies in treating solid tumors

Aspect	CAR-MΦ	CAR-T	CAR-NK
Mechanism of Action	Phagocytosis of tumor cells; secretion of pro-inflammatory cytokines; antigen presentation.	Direct cytotoxicity through antigen-specific T cell activation.	Direct cytotoxicity; release of cytolytic granules; ADCC.
Efficacy in Solid Tumors	Promising, especially in modulating the TME and overcoming immunosuppression.	Limited efficacy due to immunosuppressive TME, difficulty in tumor infiltration, and antigen escape.	Emerging efficacy; faces challenges like CAR-T but with the added benefits of innate targeting mechanisms.
Challenges	Identifying specific tumor antigens for targeting; managing CRS.	Immunological barriers like T-cell exhaustion, antigen loss variation, and immunosuppressive TME.	Overcoming immunosuppressive TIME; ensuring persistence and sustained activity in the hostile TME.
Potential Benefits	Better infiltration into tumors; ability to remodel the TME; potential for sustained antitumor immunity.	High specificity and potency in hematological malignancies; potential for rapid and complete responses.	Lower risk of causing GVHD; potential for quicker and more natural immune response.
Clinical Application	Early clinical trials showing promising results; potential for use in combination therapies.	Well-established in certain hematologic cancers; expanding into trials for solid tumors.	Fewer clinical trials than CAR-T; potential for combination with other immunotherapies for solid tumors.

In conclusion, while CAR-MΦ offers unique advantages in cancer therapy through their phagocytic and antigen-presenting abilities and their capacity to modulate the TME, significant hurdles remain [33, 35]. The challenges of targeting specificity and managing CRS, along with unanswered questions about improving the specificity of CAR-MΦ for tumor cells, enhancing their persistence in the TME, and developing effective combination therapies, continue to shape future research directions [35, 67]. Overcoming these obstacles through innovative research and development will be crucial for fully realizing the therapeutic potential of CAR-MΦ and broadening their clinical application across diverse cancer types [32]. Addressing these issues through continued research and clinical trials is essential for advancing CAR-MΦ therapy from a promising experimental approach to a robust, clinically viable treatment option for cancer [30].

## Tumor microenvironment interaction

### Immunosuppressive TME

**Fig. 2:**
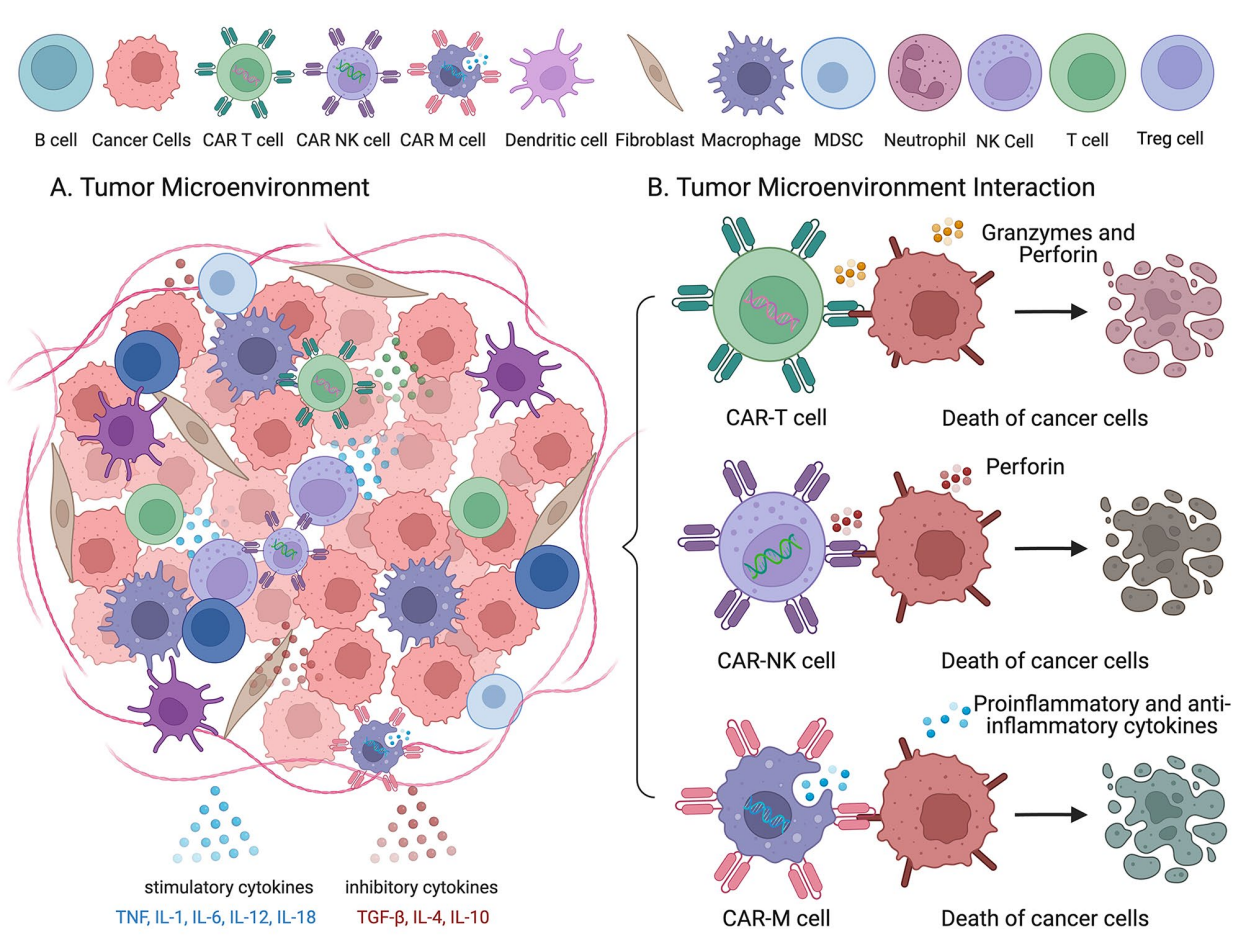
Tumor Microenvironment Interaction. This figure illustrates the interactions of various immune cells within the TME and their mechanisms for targeting cancer cells. The top row includes legends for different cell types. The central section depicts a dense network of cancer cells interspersed with various immune cells within the TME, highlighting stimulatory cytokines, including TNF, IL-1, IL-6, IL-12, and IL-18 that enhance immune responses, and inhibitory cytokines such as TGF-β, IL-4, and IL-10 that suppress immune responses. CAR-T cells attack cancer cells by releasing granzymes and perforin, leading to cell death. CAR-NK cells kill cancer cells through direct cytotoxicity using perforin. CAR-MΦ cells, with their dual role, kill cancer cells by secreting pro-inflammatory cytokines and presenting antigens. The death of cancer cells post-interaction with these CAR cells emphasizes their respective mechanisms of action. This figure underscores the complexity of the TME, and the strategies employed by CAR-T, CAR-NK, and CAR-MΦ cells to overcome immunosuppressive barriers and effectively target cancer cells

The TME significantly impacts the efficacy of adoptive cell therapies such as CAR-T and CAR-NK therapies. The TME’s complex array of cellular and molecular components creates a hostile environment that challenges the therapeutic success of these innovative cancer treatments [38, 69]. Figure 2 illustrates the interactions of various immune cells within the TME and their mechanisms for targeting cancer cells.

The immunosuppressive nature of the TME notably hinders the effectiveness of both CAR-T and CAR-NK cells [8, 84]. While CAR-T cells have achieved remarkable success in hematologic cancers, their transition to treating solid tumors is fraught with difficulties due to substantial physical and biochemical barriers. These barriers include dense extracellular matrices that impede cell infiltration and various immunosuppressive cells and cytokines that restrict access to tumor cells and promote T-cell exhaustion, reducing their cytotoxic functions [5, 69].

Similarly, despite their innate ability to recognize and kill tumor cells without prior sensitization, CAR-NK cells encounter limitations within the TME that affect their persistence and cytotoxic activity. The suppressive factors within this environment can deactivate their natural cytotoxic mechanisms and reduce their overall effectiveness against tumors [84, 85].

Efforts to mitigate the effects of the TME on CAR therapies involve consensus-driven and innovative strategies [86, 87]. One common approach is engineering CAR cells to express cytokines that counteract the TME’s suppressive nature [45, 88]. For instance, incorporating genes that encode stimulatory cytokines such as IL-12 or IL-15 aims to maintain their antitumor activity within this challenging environment [89, 90].

The use of checkpoint inhibitors alongside CAR therapies is also gaining traction. These inhibitors can block the pathways tumors use to suppress immune responses, potentially rejuvenating exhausted CAR-T cells and boosting their functionality within the TME [91, 92].

Overcoming the physical barriers within the TME is crucial for the success of these therapies [93, 94]. Innovations such as enzymatic degradation of the extracellular matrix and employing nanoparticles for more effective delivery of CAR cells are being explored to enhance their infiltration and persistence in tumor sites [95, 96].

Despite significant advances, substantial gaps remain in understanding how to adapt CAR therapies effectively for solid tumors [67, 68, 70]. Questions persist about the optimal design of CAR constructs to improve their affinity for antigens and resistance to immunosuppressive cytokines [15, 97]. Furthermore, understanding the long-term effects of using stimulatory cytokines within CAR constructs on the systemic immune response and patient safety is crucial [12, 98].

As research continues to evolve, filling these gaps will be vital for enhancing the clinical applicability and success of CAR therapies in treating solid tumors. This ongoing exploration is critical to improving outcomes for patients facing these challenging conditions [67, 99].

### CAR-MΦ in the TME

CAR-MΦ offers a transformative strategy in the evolution of adoptive cell therapies, targeting the intricate dynamics of the TME [100]. These engineered macrophages aim to reprogram tumor-associated macrophages (TAMs), which tumors typically manipulate to support cancer growth and suppress immune responses [13, 101].

By integrating CAR constructs into macrophages, researchers aspire to transform these generally suppressive immune cells into potent anti-tumor agents [32]. CAR-MΦ is engineered to recognize and destroy tumor cells, potentially reversing the immunosuppressive functions of TAMs and converting them into cells that actively bolster immune responses against the tumor [19, 102]. This approach, however, is subject to significant debate. While some studies have shown promising results with successful reprogramming leading to tumor regression, others point out the variability of TAM behavior across different tumor types and stages, which can critically affect the outcomes of CAR-MΦ therapies [25, 103].

In addition to reprogramming, CAR-MΦ exhibits a unique potential for beneficial interactions with other immune cells within the TME, such as T cells and NK cells [32]. These interactions, which involve antigen presentation and co-stimulation, could significantly enhance T-cell activation and immune response against tumors. Moreover, the ability of CAR-MΦ to assist NK cells might amplify natural cytotoxic responses against the tumor [104]. Despite these theoretical advantages, the effectiveness and consistency of these interactions in vivo remain a topic of ongoing research, with studies reporting variable outcomes depending on the specific conditions of the TME.

Another promising aspect of CAR-MΦ therapy is its potential synergy with checkpoint inhibitors [105]. These inhibitors, designed to block the proteins that tumors use to shut down immune responses, could be particularly effective when combined with CAR-MΦ, potentially sustaining their activation and tumor-killing ability within the typically immunosuppressive TME [106]. While there is general agreement on the potential benefits of this combination, the empirical evidence is still accumulating, and the optimal strategies for their use continue to be debated.

Despite significant advances, several critical gaps remain in understanding CAR-MΦ’s role within the TME [19, 107]. Questions about the efficacy of TAM reprogramming in various types of solid tumors, the long-term effects of CAR-MΦ therapy on the immune system and tumor dynamics, and the optimal strategies for combining CAR-MΦ therapy with other treatments are crucial for designing a more effective therapeutic strategy [108]. Additionally, understanding how CAR-MΦ navigates the complex regulatory pathways within the immune system and identifying targets to enhance their persistence and efficacy are vital areas needing further exploration [8].

Addressing these gaps through comprehensive research and controlled clinical trials will be essential for advancing CAR-MΦ therapy from a promising experimental approach to a robust, clinically viable treatment option across various cancers. As the field evolves, these efforts will be crucial in optimizing the design and clinical application of CAR-MΦ in oncology [17, 34].

## Mechanisms of action

### Antigen recognition and activation pathways

CAR-MΦ represents a pivotal shift in cancer immunotherapy, incorporating engineered antigen recognition and activation pathways that distinguish them from traditional CAR-T and CAR-NK cells. These pathways are crucial for optimizing CAR-MΦ therapies for clinical use [17].

CAR-MΦ is engineered with synthetic receptors targeting specific tumor antigens. These receptors typically include an extracellular antigen-binding domain derived from an antibody’s single-chain variable fragment (scFv) connected to intracellular signaling domains that trigger macrophage activation and effector functions upon antigen engagement [34]. The selection of signaling domains remains a subject of considerable debate as researchers seek to optimize configurations that maximize therapeutic benefits without provoking excessive inflammatory responses [8, 19, 109].

### Phagocytosis and antigen presentation

The process of tumor cell engulfment by CAR-MΦ involves intricate biological mechanisms [17, 110]. CAR-MΦ, equipped with engineered receptors, binds explicitly to antigens expressed on tumor cells [20, 34]. This binding triggers phagocytic activity, leading to tumor cell internalization and degradation within phagolysosomes [111].

**Fig. 3:**
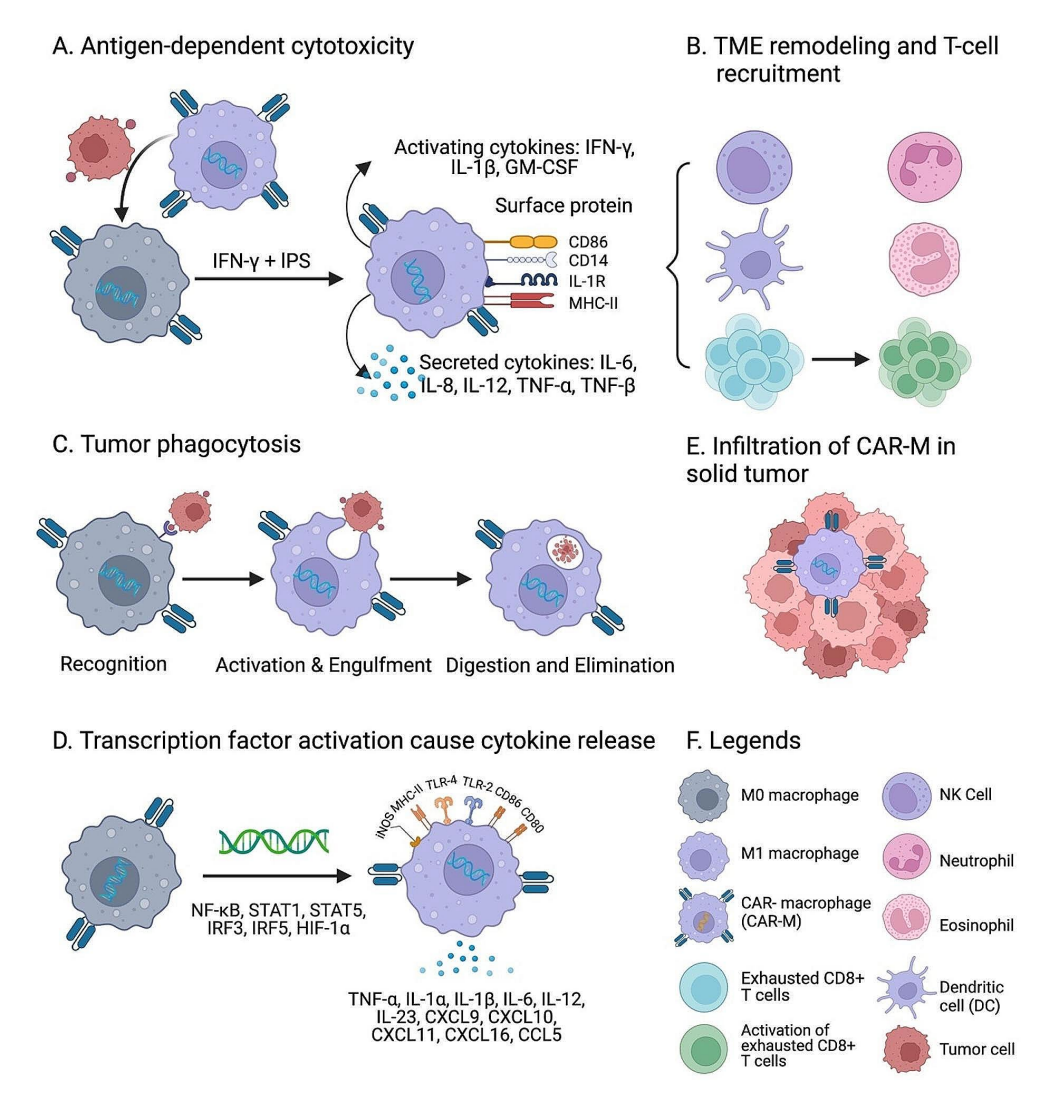
Mechanisms of Action of CAR-MΦ in the TME. This figure illustrates the multifaceted mechanisms through which CAR-MΦ exert their effects within the TME: (**A**) Antigen Recognition and Activation Pathways: CAR-MΦ are equipped with engineered receptors that target specific tumor antigens and intracellular signaling domains, allowing them to switch from an M0 state to an M1 state, which is pro-inflammatory and antitumor. (**B**) TME Remodeling: CAR-MΦ can remodel the TME by releasing pro-inflammatory cytokines that activate exhausted CD8^+^ T cells and other innate immune cells, including NK cells, dendritic cells, eosinophils, and neutrophils. (**C**) Tumor Phagocytosis: When tumor antigens bind to the CAR receptor on the surface of CAR-MΦ, activation signals are generated, leading to tumor phagocytosis. This process includes recognition, activation, engulfment, and elimination within phagolysosomes. (**D**) Transcription Factor Activation and Cytokine Release: CAR-MΦ activation involves transcription factors like NF-kB, releasing inflammatory cytokines that can activate T cell-mediated immunity against tumors. (**E**) Infiltration of CAR-MΦ in Tumor Cells: CAR-MΦ play vital roles in the TME and, through their direct effects, efficiently eliminate tumor cells by phagocytosis and antigen presentation to CD8^+^ T cells, bridging innate and adaptive immunity. (**F**) Legend: The legend shows the names of immune and tumor cells

The role of CAR-MΦ in antigen cross-presentation to T cells is central to their functionality, bridging innate and adaptive immunity [112]. After processing, peptides derived from tumor cells are presented via MHC class I molecules, crucial for activating CD8^+^ cytotoxic T cells [113, 114]. This step initiates a broader immune response, allowing T cells to recognize and destroy other tumor cells expressing the same antigens. Debates persist about its efficiency and reliability across different tumor environments [115, 116]. Figure 3 illustrates the various mechanisms through which CAR-MΦ exert their effects within the TME, highlighting their multifaceted approach to tumor eradication [30].

Despite advancements, gaps remain in understanding CAR-MΦ’s phagocytosis and antigen presentation [117, 118]. Questions about the efficiency of tumor cell engulfment within an immunosuppressive TME and factors enhancing this process persist. Additionally, the effectiveness of antigen presentation varies across different patients and tumor types, raising concerns about consistency [119, 120]. The variability of the TME significantly influences CAR-MΦ’s ability to perform effectively, necessitating strategies to overcome these challenges [44, 94].

Addressing these gaps is crucial for CAR-MΦ therapy advancement [33]. Investigating molecular mechanisms that enhance CAR-MΦ and T-cell interactions, optimizing CAR constructs for improved antigen presentation, and devising methods to counteract TME immunosuppressive barriers are essential for future research [88, 121]. A deeper understanding of these processes is vital for enhancing CAR-MΦ therapy design and clinical application [30, 32, 122].

### Cytokine secretion and immune activation

CAR-MΦ impacts cancer immunotherapy by secreting key cytokines that activate and orchestrate the immune response [22, 123]. These cytokines facilitate local and systemic anti-tumor actions, which are crucial for therapeutic success [45, 88, 124].

Key cytokines like IL-12, IL-23, and TNF-α are central to immune modulation [44, 124]. IL-12 activates NK cells and drives CD4^+^ T cells into Th1 cells, which produce IFN-γ, critical for antitumor immunity [125]. IL-23 supports Th17 cell proliferation, which can support or suppress tumor growth depending on the context [126].

The ability of these cytokines to recruit and activate other immune cells is pivotal [19, 39, 124]. Chemokines such as CCL2 and CCL5 attract immune cells to the tumor site, facilitating a robust immune attack, critical for combating tumor heterogeneity and adaptive resistance mechanisms [13, 127, 128].

While the theoretical benefits of cytokine-mediated immune recruitment and activation are acknowledged, debates persist about optimal cytokine levels and types [129, 130]. Excessive cytokine secretion can lead to systemic inflammation and side effects, necessitating careful modulation in CAR-MΦ design [107, 131].

Significant gaps remain in understanding the precise mechanisms of CAR-MΦ cytokine secretion and immune response modulation [132, 133]. Further research is needed to optimize cytokine profiles for therapeutic efficacy and safety, particularly in solid tumors [17, 35].

### Phenotypic characterization of CAR-MΦ

Phenotypic characterization of CAR-MΦ is essential to understand their transitions from an M0 (naive) state to an M1 (pro-inflammatory) or M2 (anti-inflammatory) state. The characterization involves assessing the expression of surface markers, cytokine profiles, and functional properties of the engineered macrophages. This incorporation of co-stimulatory domains such as CD28 or 4-1BB in the CAR construct is crucial for macrophage activation, survival, and functionality [19].

### Tumor cell killing mechanisms

CAR-MΦ targets cancer cells through direct and indirect mechanisms, showcasing their multifaceted role in cancer therapy [19, 33, 35, 134]. Directly, CAR-MΦ engages in phagocytosis, binding to tumor antigens and initiating tumor cell engulfment and degradation within phagolysosomes [8, 110]. This direct interaction physically removes tumor cells and leads to their breakdown and destruction, a process noted for its effectiveness in eliminating tumor cells [20, 135].

Indirectly, CAR-MΦ alters the TME through immune modulation. Secreting cytokines and presenting tumor antigens activate and recruit immune cells to the tumor site, enhancing the overall immune response [36, 102]. This recruitment strategy is critical for immediate efficacy and sustaining long-term anti-tumor activity [30, 136].

Despite recognized benefits, gaps remain in understanding CAR-MΦ’s capabilities [19, 22]. Questions about phagocytic efficiency in immunosuppressive environments and optimal cytokine profiles for sustained immune responses persist [17, 35]. Further research is required to optimize CAR-MΦ designs for consistent clinical outcomes [30, 32].

### Strategies for enhancing CAR-MΦ efficacy

Advancing through genetic engineering, researchers refine CAR constructs to improve macrophage activation specificity and durability. Innovations like switch receptors and signaling pathway modifications fine-tune anti-tumor effects and control immune responses [137, 138]. These advancements aim to amplify CAR-MΦ’s capabilities while managing off-target effects and systemic toxicity [139, 140].

Exploring combination therapies adds complexity and promise. CAR-MΦ is used alongside other immunotherapeutic agents, like checkpoint inhibitors, designed to overcome TME immunosuppressive barriers and enhance immune response [19, 30, 34, 136]. Combining CAR-MΦ with traditional treatments like chemotherapy and radiation aims to reduce tumor burden and modify the TME for more effective CAR-MΦ activity [36, 88, 141].

Debates continue over the best combination methods, treatment timings, and managing compounded side effects [142, 143]. Substantial gaps remain in understanding the long-term efficacy and safety of these strategies, their impact on patient outcomes, and optimal CAR-MΦ integration with existing treatments. Continuous innovation and rigorous clinical testing are crucial for transitioning CAR-MΦ therapies from experimental approaches to standard cancer care, enhancing direct anti-tumor activities and systemic immune responses [8, 35, 144].

Currently, CT-0508 is safe and feasible to manufacture. Early data demonstrate trafficking, TME modulation, and potential antitumor T cell immunity induction. The study is actively enrolling participants [18]. We look forward to the results from the ex vivo combination sub-study with pembrolizumab and the continued development of CAR-MΦ and CAR-Monocyte therapies.

## Technological and manufacturing challenges

### Optimization of CAR-MΦ design

The Optimization of CAR-MΦ design is crucial in addressing the challenge posed by the variety of expressions on tumor cells. The presence of varying levels or types of antigens among tumor cells within a tumor mass or across tumors can hinder the effectiveness of CAR-MΦ therapies if the engineered receptors target only specific antigens present in certain tumor cell subsets.

One approach to tackle variability is designing CAR-MΦ that can target multiple antigens simultaneously. By incorporating single-chain variable fragments (scFvs) into the CAR structure, these CAR-MΦ can identify and bind to various TAAs. This multi-targeting strategy increases the chances of reaching a range of tumor cells within heterogeneous tumors [17, 19].

Another strategy involves utilizing scFvs that recognize epitopes shared by an array of tumor cells. These reactive scFvs are created to bind to antigens found across different types of tumors, thereby enhancing the overall effectiveness of CAR Macrophage therapy against heterogeneous tumors [122].

Furthermore, CAR-MΦ can be designed with signaling domains that allow them to adjust their response according to the specific conditions in the TME. For example, including stimulatory molecules, like CD28 or 4-1BB, in CAR design improves macrophage survival, growth, and ability to engulf particles even when encountering different antigen expression levels [8].

Pairing CAR-MΦ therapy with treatments such as checkpoint inhibitors or traditional chemotherapies can tackle the challenge of antigens. By disrupting the immune-suppressing tumor microenvironment and reducing the diversity of tumor cells, these combinations can boost CAR-MΦ effectiveness in targeting a range of tumor cell populations [30, 34].

Moreover, recent progress in epigenetic alterations allows for the modification of tumor cells to display antigens. Techniques like CRISPR/Cas9 can modify tumor cell genomes to make them more identifiable, to CAR-MΦ by standardizing antigen expression throughout the tumor mass [145].

**Fig. 4:**
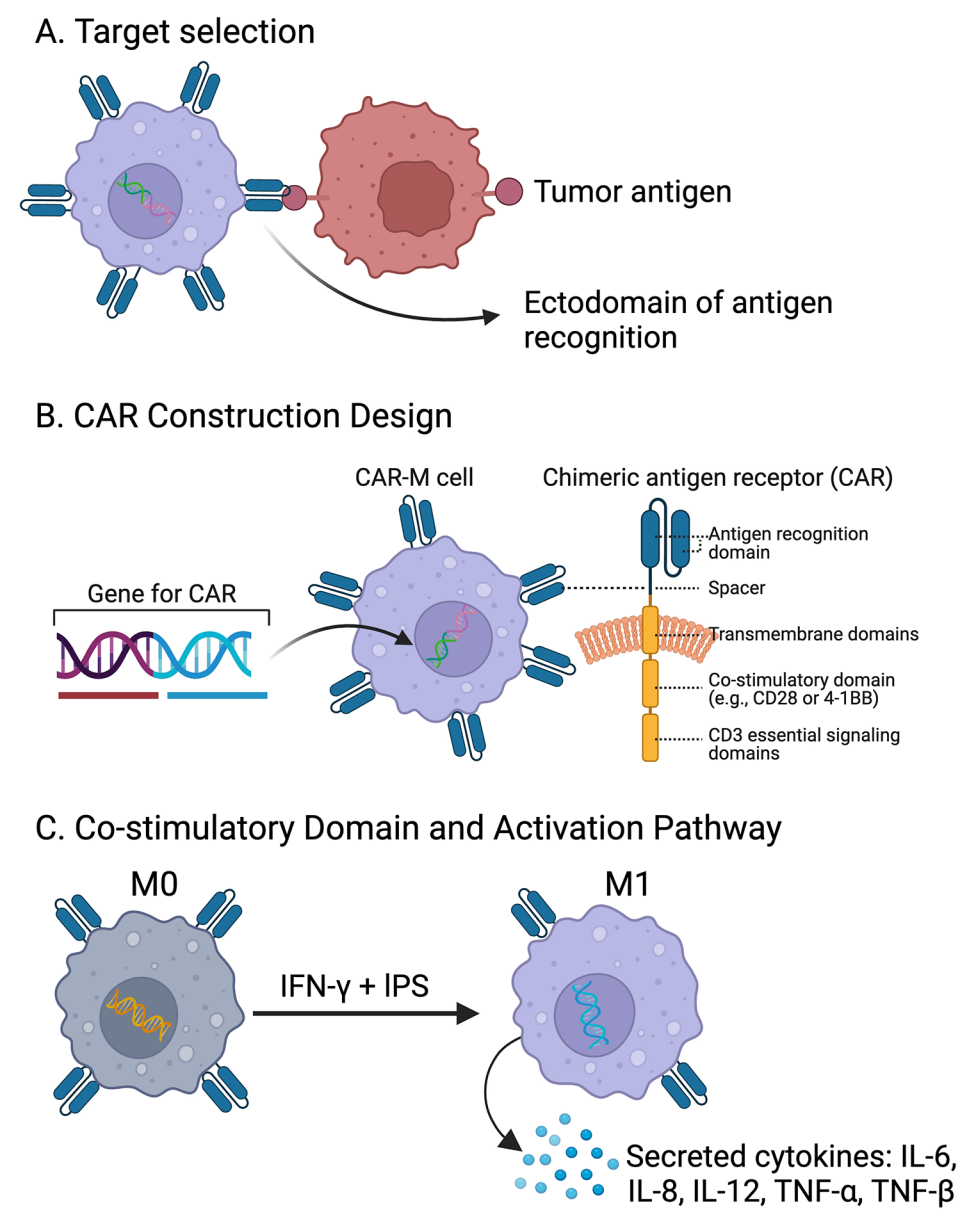
Optimization of MΦ Design. (**A**) Target Selection: CAR-MΦ is engineered to target specific tumor-associated antigens. The ectodomain of the CAR is designed to recognize these tumor antigens, ensuring precise targeting and engagement with tumor cells. (**B**) CAR Construction Design: Constructing CAR- MΦ involves inserting genes for the CAR into macrophage cells. The CAR structure includes an antigen recognition domain, a spacer, transmembrane domains, co-stimulatory domains (e.g., CD28 or 4-1BB), and CD3 essential signaling domains. These components are crucial for the activation and function of CAR-MΦ. (**C**) Co-stimulatory Domain and Activation Pathway: Upon activation by IFN-γ and IPS, CAR-MΦ transitions from an M0 (naive) state to an M1 (pro-inflammatory) state. This activation leads to the secretion of inflammatory cytokines such as IL-6, IL-8, IL-12, TNF-α, and TNF-β, which are essential for enhancing the antitumor immune response

Figure 4 provides an overview of the steps involved in optimizing CAR-MΦ design, including target selection, CAR construction design, and co-stimulatory domain activation pathways.

### Ex vivo Manufacturing processes

The ex vivo manufacturing processes for CAR-MΦ are crucial for producing compelling and consistent therapeutic cells. Differentiating and expanding macrophages under controlled conditions involves several vital factors [26]. Initially, monocytes are isolated from PBMCs of the patient or donor. These monocytes are then cultured in the presence of specific growth factors, such as M-CSF or GM-CSF, to promote their differentiation into macrophages [146]. Careful monitoring of the culture environment, including temperature, pH, and oxygen levels, is essential to maintain cell viability and functionality [147].

Regarding sourcing macrophages, the choice between autologous and allogeneic sources remains a subject of ongoing debate. Autologous macrophages, derived from a patient’s cells, are favored for their lower risk of eliciting an immune response. Yet, their use is hindered by variability in cell quality and scalability challenges [148, 149]. Conversely, allogeneic macrophages, sourced from donors, offer advantages in scalability and consistency but come with an increased risk of immune rejection and complications like GVHD [19, 38].

Transduction with viral vectors encoding the CAR construct ensures stable expression of CAR on the macrophage surface. The transduction efficiency and expression levels are rigorously evaluated using flow cytometry and molecular techniques [17]. Following transduction, the CAR-MΦ is expanded in vitro under optimized conditions supporting their growth and activation, including cytokines like IL-4 and IFN-γ for a pro-inflammatory phenotype conducive to anti-tumor activity. Validating functionality involves assessing antigen recognition, phagocytic ability, and cytokine secretion profile [150].

The protocols for differentiating and expanding macrophages are equally critical. Maintaining controlled conditions promotes the differentiation of progenitor cells into macrophages and ensures these cells appropriately express CAR constructs targeting specific tumor antigens [151]. However, balancing practical CAR expression and maintaining macrophage functionalities present a considerable challenge, often leading to variability in therapeutic outcomes [152]. High levels of CAR expression may enhance antigen recognition and tumor cell killing. However, they can lead to excessive activation and cytokine release, increasing the risk of adverse effects like CRS [142, 153]. To mitigate this, fine-tuning the transduction protocols to achieve an optimal expression level that maximizes therapeutic benefits while minimizing toxicity is necessary [154].

Quality control and standardization are pivotal for the safety and efficacy of CAR-MΦ therapies [155]. Stringent testing protocols assess the purity, potency, and identity of CAR-MΦ batches. Significant gaps exist in standardization processes, particularly concerning the long-term stability and functional consistency of CAR-MΦ post-cryopreservation, and developing universal standards applicable across different manufacturing facilities [156].

These areas of active research and debate illuminate the factors that influence the ex vivo production of CAR-MΦ. Addressing these gaps, particularly in standardizing processes and enhancing cell source viability, is crucial for advancing CAR-MΦ therapies from experimental stages to reliable clinical applications [39, 157].

### Composition of CT-0508

The CT-0508 consists of autologous macrophages genetically engineered to express a CAR that targets the HER2 expression in solid tumors. This CAR construct, in CT 0508 includes a domain with a scFv, which is specific to the HER2 antigen, and inner signaling domains like CD28 and CD3ζ that are essential for activating, sustaining, and enhancing the macrophage’s functions [141].

To genetically modify the macrophages, a viral vector is employed to insert the CAR gene into their makeup to ensure its presence on the cell surface. These elements showcase the engineering involved in CT 0508 to enhance the accuracy and efficacy of CAR MΦ therapy, for treating HER2 positive cancers [120].

### In vivo reprogramming approaches

The exploration of in vivo reprogramming approaches for CAR-MΦ centers on the advancements and challenges associated with nanoparticle-mediated delivery, as well as viral and non-viral gene editing techniques [153].

Nanoparticle-mediated delivery is emerging as a promising method for the targeted transformation of macrophages into CAR-MΦ directly within the patient’s body [158]. This technique leverages the unique capabilities of nanoparticles to deliver genetic materials or modulatory substances, especially to macrophages at tumor sites [159]. The precision of this method aims to enhance CAR constructs’ integration and functional efficacy in vivo [160]. However, there remains a debate over the consistency and safety of nanoparticle delivery, with concerns about off-target effects and the long-term viability of reprogrammed macrophages [161, 162].

Regarding gene editing, viral vectors such as lentiviruses and adenoviruses have demonstrated high efficiency in gene delivery and are widely utilized despite potential risks such as insertional mutagenesis and eliciting immune responses [163, 164].

Due to these risks, the field is somewhat divided on the reliance on viral vectors [165]. In contrast, non-viral methods like CRISPR-Cas9 and transcription activator-like effector nucleases (TALENs) offer a safer alternative, minimizing risks of genomic alterations and adverse immune reactions [166]. These non-viral techniques provide precise editing tools that can enhance the specificity of CAR-MΦ therapy. However, their efficiency and the durability of gene edits in clinical settings continue to be areas of intense investigation [167].

The literature reflects broad consensus on the potential of these in vivo reprogramming approaches to revolutionize CAR-MΦ therapies by improving their adaptability and patient-specific efficacy [39]. However, significant gaps in knowledge exist, particularly concerning the long-term effects of in vivo reprogrammed CAR-MΦ, the control of gene editing tools within complex tumor environments, and the overall safety of these interventions [8, 30, 39]. Further research is needed to address these challenges, aiming to refine these techniques for safer and more effective clinical applications.

### Cost and scalability issues

The transition of CAR-MΦ therapies from experimental to widely available treatments hinges significantly on resolving cost and scalability issues [168]. Current knowledge indicates that the high manufacturing costs stem from intricate cell engineering, complex culture conditions, and the necessity for stringent quality control, which drive up production expenses [169]. Efforts to address these costs focus on refining manufacturing techniques to enhance the efficiency of cell expansion and gene editing, which could substantially reduce costs.

However, considerable debate remains over the best methods to scale production without compromising the quality and efficacy of CAR-MΦ therapies [170]. Some consensus exists around the potential of automated bioreactors and closed-system cell culture technologies, which promise to increase production capacity and reduce labor costs and contamination risks [34, 171].

Despite these advancements, significant gaps in our understanding of scalable CAR-MΦ production persist [32]. Questions about best standardizing production protocols to ensure consistent quality across different manufacturing sites are still unresolved. Furthermore, the economic viability of scaling up CAR-MΦ therapies to meet global demand, particularly for widespread diseases like cancer, remains a contentious issue [32, 172]. Additional research and development are needed to create cost-effective, scalable manufacturing solutions to support the widespread clinical use of CAR-MΦ therapies.

## Regulatory and ethical issues

### Regulatory pathways for CAR-MΦ approval

The regulatory approval process for CAR-MΦ is an evolving area that reflects the complexities inherent in bringing new cellular therapies to market [8, 20]. While regulatory frameworks for CAR-T cell therapies provide a foundation, the unique properties of CAR-MΦ necessitate specific considerations. These include their multifunctional role in immune modulation and tissue repair, which could have different implications for patient safety and therapeutic outcomes [30, 173].

Comparatively, the regulatory journey for CAR-T cells has established a precedent that emphasizes stringent evaluation of safety and efficacy. However, CAR-MΦ therapies introduce new variables, such as their phagocytic nature and the broad spectrum of cytokine production, which can affect both tumor and non-tumor tissues [158]. This raises debates about the adequacy of existing regulatory pathways to fully address the nuanced risks associated with macrophage-based therapies.

Controversies emerge particularly around the long-term effects of CAR-MΦ, given their potential to extensively alter immune system dynamics [30, 36]. Regulatory bodies are challenged to develop guidelines that adequately address these concerns while fostering the innovation necessary to realize CAR-MΦ’s therapeutic potential [174]. There is consensus on the need for tailored regulatory approaches that consider the unique biological behaviors of macrophages and their interaction with the TME.

However, significant gaps in knowledge persist, especially regarding the long-term safety and behavior of genetically modified macrophages in humans. These gaps highlight the need for comprehensive preclinical and clinical data to inform regulatory decisions, ensuring that CAR-MΦ therapies are both practical and safe for patients. This section delves into the current state of regulatory processes, emphasizing the ongoing dialogue between researchers, regulators, and the biopharmaceutical industry to refine the approval pathways for these promising but complex therapies.

### Safety monitoring and reporting

Safety monitoring and reporting for CAR-MΦ therapies are critical components of their clinical development, given the significant potential for adverse effects such as CRS and other immune-related events. Current frameworks for managing these risks involve protocols adapted from CAR-T cell therapies but tailored to address the unique properties of macrophages. The protocols emphasize early detection and intervention to mitigate the severity of CRS, which remains a primary concern with all CAR therapies [87, 175].

There is a consensus on the need for robust, long-term follow-up to monitor the late-onset effects of CAR-MΦ treatments, which are not fully understood due to these therapies’ novel mechanisms of action [176]. The long-term safety profile is especially pertinent given the CAR-MΦ’s ability to alter the TME and potentially affect the immune system in unforeseen ways.

Debates continue over the best practices for safety monitoring, particularly concerning the balance between thorough data collection and the practicality of long-term follow-up in a clinical setting [5, 177]. Questions also persist about the sufficiency of current adverse event reporting systems and whether they adequately capture the range of possible complications, particularly those unique to macrophage-based therapies [160].

Significant gaps in knowledge remain, particularly in how CAR-MΦ interacts with diverse patient immunology over extended periods [142]. Further research is needed to develop and standardize safety monitoring protocols that can effectively track and manage the complex safety profile of CAR-MΦ therapies [178]. These efforts are crucial for ensuring patient safety and facilitating the broader adoption of this promising therapeutic approach in oncology.

### Ethical considerations

The integration of CAR-MΦ therapies into clinical practice brings forth complex ethical considerations, particularly regarding patient selection, informed consent, and equitable distribution of these emerging treatments [179]. The current discussion focuses on ensuring ethical standards in patient selection by establishing scientifically valid and morally sound criteria, aiming to balance the potential benefits and risks associated with CAR-MΦ therapies effectively [180]. The informed consent process is critical, as it must fully educate patients about the experimental nature of CAR-MΦ, potential risks, expected benefits, and possible side effects to ensure decisions are made with adequate knowledge and free of coercion [181].

Debates around access and equity are particularly vigorous, reflecting broader concerns about the availability of cutting-edge medical treatments. There is consensus on the need for strategies to prevent socioeconomic status or geographic location from limiting access to these therapies. However, there is controversy over how best to implement such strategies effectively and relatively [182]. The literature highlights a significant gap in frameworks that could guide equitable access, suggesting that international collaboration is needed to develop policies that facilitate broad and fair distribution without compromising the quality of care.

The ethical implications of CAR-MΦ therapies also extend to long-term societal impacts, such as the potential for altering healthcare paradigms and patient expectations. Current ethical discussions often do not fully address the long-term consequences of widespread CAR-MΦ adoption, indicating a critical area for future research and policy development [183]. As CAR-MΦ technologies advance, ongoing ethical scrutiny will be essential to navigate the complexities of introducing these innovative therapies into routine clinical settings, ensuring they benefit all patients regardless of their background.

## Conclusion and future perspectives

This review has critically analyzed the evolving field of CAR-MΦ therapies, identifying groundbreaking advancements and persistent challenges in their development. The synthesis of current research underscores CAR-MΦ as a pioneering approach within cancer immunotherapy, particularly for solid tumors where conventional CAR-T therapies face limitations. Key findings reveal that while CAR-MΦ demonstrates significant potential in modulating the TME and enhancing immune responses, there are substantial gaps in optimizing CAR constructs for maximum specificity and efficacy [35].

Debate continues over the best strategies for CAR-MΦ deployment, with discussions centering on the balance between potent anti-tumor actions and controlling systemic immune reactions to prevent adverse effects. The literature reflects a consensus on the innovative capacity of CAR-MΦ to transform cancer treatment. Yet, it also highlights controversies regarding their long-term efficacy and safety, which remain inadequately explored in diverse clinical settings [32, 136].

Future research should address these gaps by refining genetic engineering techniques to enhance the precision and stability of CAR-MΦ activation [144]. Expanding clinical trials to include more comprehensive range of tumor types and patient demographics is crucial for understanding the broader applicability of CAR-MΦ therapies [35]. Additionally, ethical considerations regarding patient selection and access to these emerging therapies need a thorough examination to ensure equitable treatment across different populations [183]. By continuing to explore these areas, the field can move towards fully integrating CAR-MΦ into the next generation of standard cancer care, potentially revolutionizing outcomes for patients with previously resistant forms of cancer [19].

### Electronic supplementary material

Below is the link to the electronic supplementary material.

Supplementary Material 1

## Data Availability

No datasets were generated or analysed during the current study.

## Abbreviations

ACT: adoptive cell transfer
ADCC: antibody-dependent cellular cytotoxicity
ALL: acute lymphoblastic leukemia
CAR: chimeric antigen receptor
CAR-MΦ: CAR Macrophage
CAR-NK: CAR natural killer cell
CAR-T: CAR T cell
CRS: cytokine release syndrome
DLBCL: diffuse large B-cell lymphoma
HER2: human epidermal growth factor receptor 2
HLH: hemophagocytic lymphohistiocytosis
ICANS: immune effector cell-associated neurotoxicity syndrome
IFN-g: Interferon gamma
ICI: immune checkpoint inhibitor
IL: interleukin
MAS: macrophage activation syndrome
NK: natural killer
TALENs: transcription activator-like effector nucleases
TAMs: tumor-associated macrophages
TME: tumor microenvironment
TNF-a: tumor necrosis factor-alpha
